# Pretreatment with Pectoral Nerve Block II Is Effective for Reducing Pain in Patients Undergoing Thoracoscopic Lobectomy: A Randomized, Double-Blind, Placebo-Controlled Trial

**DOI:** 10.1155/2021/6693221

**Published:** 2021-04-16

**Authors:** Ge Luo, Jianjun Zhu, Huadong Ni, Qinghe Zhou, Yaping Lu, Qihong Shen, Yibing Yao, Qiuli He, Jie Fu, Ming Yao

**Affiliations:** ^1^Department of Anesthesiology, Bengbu Medical College, Bengbu, China; ^2^Department of Anesthesiology and Pain Research Center, The First Hospital of Jiaxing or the Affiliated Hospital of Jiaxing University, Jiaxing, China

## Abstract

**Background:**

Although video-assisted thoracoscopy has a smaller incision than traditional surgery, the postoperative pain is still severe. Ultrasound-guided pectoral nerve block (PECS) II is a new technique that can reduce pain in patients, and it had not been reported in the analgesia after thoracoscopic lobectomy.

**Methods:**

40 patients scheduled for thoracoscopic lobectomy were randomly divided into two groups. Patients in the PECS II group received 0.5% ropivacaine 25 ml before the general anesthesia, while patients in the placebo group received 0.9% saline. Thirty minutes after the block was performed, a pin-prick test was used to analyze the sense of pain of T2-T6 segments. The primary endpoint was the total consumption of fentanyl. Data were collected in the postanesthesia care unit (PACU) and in the ward within 24 hours after operation.

**Results:**

The total consumption of fentanyl and the consumption of fentanyl in the intravenous analgesia pump within 24 hours after the operation were significantly lower in the PECS II group compared to the placebo group (*p* < 0.05). The implementation rate of rescue analgesia during operation and in PACU in the PECS II group was significantly lower than that in the placebo group (*p* < 0.05). The numerical rating scale (NRS) in 1 and 4 h after operation was lower in the PECS II group (*p* < 0.05). Mean arterial pressure (MAP) and heart rate (HR) of the PECS II group at chest entering (T1) were significantly lower than those in the placebo group (*p* < 0.05).

**Conclusion:**

Preconditioning of PECS II can stabilize the intraoperative circulation and significantly reduce pain and the consumption of opioids after operation.

## 1. Introduction

Over recent years, video-assisted thoracic surgery (VATS) has become a widely used tool for lung surgery [1]. Some studies have pointed out that the effect of VATS on vital capacity and exercise ability is smaller than that of posterolateral thoracotomy and may shorter hospital stays [2, 3]. Although VATS results in smaller surgical incisions, studies have shown that smaller surgical incisions do not seem to mean less postoperative pain, and postoperative pain caused by VATS is still moderate to severe [4].

PECS II is a novel, safe technique developed in 2012 by Blanco and his team. According to Blanco's description, PECS II, also known as modified PECS block, is optimized on the basis of the original PECS I block technique. PECS I is a technique that blocks the lateral and medial thoracic nerves by injecting a small amount of local anesthetic between pectoralis major muscle (PMM) and pectoralis minor muscle (PmM) to achieve prethoracic area block. PECS II is a new block technique which injects local anesthetic between PmM and serratus anterior muscle (SAM) to block the lateral branch of the spinal nerve and improve the block of the lateral chest wall. At present, PECS II is widely used in perioperative pain management of breast surgery [5–7]. Particularly when lymph node dissection is needed in breast cancer surgery, compared with PECS I, the block of long thoracic nerve provided by PECS II can effectively cover the armpit and lateral chest wall area, provide good analgesic effect, and improve patients' perioperative comfort and satisfaction. Whether single-hole or multihole thoracoscopic lobectomy, the pain derived from the incision is mainly concentrated in the lateral chest wall, while PECS II provides segmental block of T2-T6 and covers nerve branches such as long thoracic nerve and intercostobrachial nerve, which provides a theoretical basis for perioperative analgesia after thoracoscopic lobectomy. However, the application of PECS II in postoperative analgesia after thoracoscopic lobectomy has not been reported.

In this study, PECS II was applied to the perioperative analgesia management of thoracoscopic lobectomy for the first time. Ultrasound-guided PECS II block provided an effective guarantee for the safety of the operation. The block method proposed by Blanco was used to inject local anesthetic into each layer of muscle successively. Considering there are no large branches of blood vessels in the puncture site, the possibility of local anesthetic poisoning and direct nerve injury is very low. We aimed to use PECS II block as preconditioning in thoracoscopic lobectomy to explore whether PECS II can stabilize the circulation during the operation, relieve the pain of the incisions after the surgery, reduce the use of opioids, and provide evidence for the safety and effectiveness of PECS II.

## 2. Methods

### 2.1. Patients

This study has been approved by the Ethics Committee of Affiliated Hospital of Jiaxing University. After providing written informed consent, forty ASAI-II patients aged 18-70 who underwent thoracoscopic lobectomy in the Affiliated Hospital of Jiaxing University between April and December 2020 were included in the study.

### 2.2. Inclusion and Exclusion Criteria

The inclusion criteria are as follows: (1) agreed to undergo thoracoscopic lobectomy; (2) volunteered to participate in this study and sign the informed consent form. The exclusion criteria are as follows: (1) congenital anatomic structure variation of trachea and bronchus; (2) severe obesity (BMI > 35 kg/m^2^); (3) ASA ≥ III; (4) moderate and severe obstructive or restrictive ventilatory dysfunction; (5) cardiac insufficiency; (6) pregnancy; (7) allergic to local anesthetic; (8) abnormality of platelet and coagulation function; (9) malformation or infection; (10) suffering mental illness; (11) short-term or long-term use of opioids; (12) neurological dysfunction; (13) unable to understand NRS and patient-controlled intravenous analgesia (PCIA) usage.

### 2.3. Grouping and Randomization

Forty patients were randomly assigned to two groups using computer-generated random numbers. The randomly assigned numbers in each group were hidden in sealed and opaque envelopes, which were then randomly distributed to every patient. The envelope was then handed to an anesthetic nurse before the operation, which prepared the solution according to the patient's number and handed the needle to the operator who was blinded to the grouping. The block was done with 25 ml 0.5% ropivacaine [5] (PECS II group) and 25 ml 0.9% saline (placebo group).

### 2.4. Application of Block Interventions

The nerve block was completed 30 min before the operation; the punctures were performed under aseptic conditions using a 22G∗50 mm nerve block needle with a high-frequency linear probe. PECS II was performed according to Blanco et al.'s method [8]. Briefly, the patient was placed in the supine position. The ultrasound probe was placed at the midclavicular level inferolaterally to locate the axillary artery and vein. The probe was then moved laterally until pectoralis minor and serratus anterior muscles were identified at the level of the third rib. The needle was advanced in the plane of the probe from medial to lateral in an oblique manner until the tip entered the plane between the pectoralis major and minor, and ropivacaine 0.5%, 10 ml was injected. After the injection was completed, the needle was continued to be injected until the needle tip reached between the pectoralis minor muscle and the anterior serratus muscle, and 0.5% ropivacaine 15 ml was injected into this area. Similarly, the placebo group was injected with the same amount of 0.9% saline.

### 2.5. Preoperative Evaluation of Regional Anesthesia

Thirty minutes after the injection of the local anesthetic, a sensory level was assessed by a blinded observer with a pin-prick test in each dermatomal distribution from T2 to T6. Then, the number of segments with lighter pain was recorded. If the pain sensation in the segment did not decrease or disappear, then the block was considered unsuccessful [5].

### 2.6. Anesthesia Application

After the circulation was stabilized, the HR and MAP at baseline (T0) were recorded. Anesthesia was induced with fentanyl 5 *μ*g/kg, propofol 2 mg/kg, and cisatracurium 0.2 mg/kg. After intubation, one-lung ventilation was performed in volume control mode. During the operation, propofol 2 mg/kg·h was intravenously injected, remifentanil 0.05 *μ*g/kg·min, and inhaled with 1.5% sevoflurane at 2 l/min of pure oxygen.

If MAP continued to exceed 20% of the baseline value within 5 min, fentanyl 25 *μ*g iv was given. The HR and MAP of entering chest (T1), specimen excision (T2), and closing chest (T3) and additional dose of fentanyl were recorded during operation. After the operation, PCIA was connected before leaving the operation room, and the formula of all pumps was the same: fentanyl 1 mg + granisetron 6 mg/100 ml. The mode of the analgesia pump was bolus only. Parameter of PCIA: bolus: 2 ml; continue: 0 ml; lock time: 15 min.

### 2.7. PACU Management

Patients were received by a nurse who did not know the contents of the study. All patients were intubated into PACU with a double lumen endobronchial tube. The static NRS was used to evaluate whether the rescue analgesia measures were required in PACU: NRS 4-6: intravenous fentanyl 25 *μ*g; (2) 7-10: fentanyl 50 *μ*g. At the same time, the NRS were recorded before the rescue analgesia. The time to first analgesic request was defined as the time of the first addition of opioids after the operation.

### 2.8. A 4 h and 24 h Follow-Up

The collection of perioperative data and postoperative follow-up (4 hours and 24 hours after the operation) was carried in the ward by a researcher who was blind to the grouping. The pain was evaluated using an 11-point NRS.

### 2.9. Outcome Measures

The primary endpoint was the total consumption of fentanyl. The secondary endpoints were as follows: (1) the dose of opioids used for rescue analgesia (consumption of fentanyl during the operation, in PACU and in the ward); (2) NRS (1 h, 4 h, and 24 h after operation); (3) time for the first analgesic request; (4) MAP and HR during T1, T2, and T3; (5) incidence of adverse effects; and (6) patient satisfaction.

### 2.10. Sample Size

The sample size was calculated based on the preexperiment, which included 16 patients with eight patients in each group. The average total consumption in the PECS II group was 22.50 ± 22.520, while in the control group was 116.88 ± 95.167. After *α* was set to 0.01, and the power to 0.80, the calculation result suggested a minimum of 16 cases. With consideration to the patients' drop-out, we increased the number of patients by 20%, finally enrolling 20 cases in each group.

### 2.11. Statistical Analysis

SPSS 25.0 (IBM, Chicago, USA) was used for statistical analysis. The Kolmogorov-Smirnov test was used to verify the normality of the data. The nonnormally distributed data were expressed by the median (IQR) and compared by the Mann-Whitney *U* test. The distribution trend of normally distributed data was described by mean ± SD and compared by the independent sample *t*-test. The counting data were compared using Pearson's chi-squared test or Fisher's exact test (depending on the specific situation). A *p* value < 0.05 was considered to be statistically significant.

## 3. Results

A total of 66 potential subjects were screened. Among these, 24 subjects were excluded because of their age, ASA grade, and refusal to join the study during the recruitment process. Each subject read and signed informed consent. All patients were randomly grouped based on a computerized randomization table created by a researcher who was not involved in this study.

**Figure 1:**
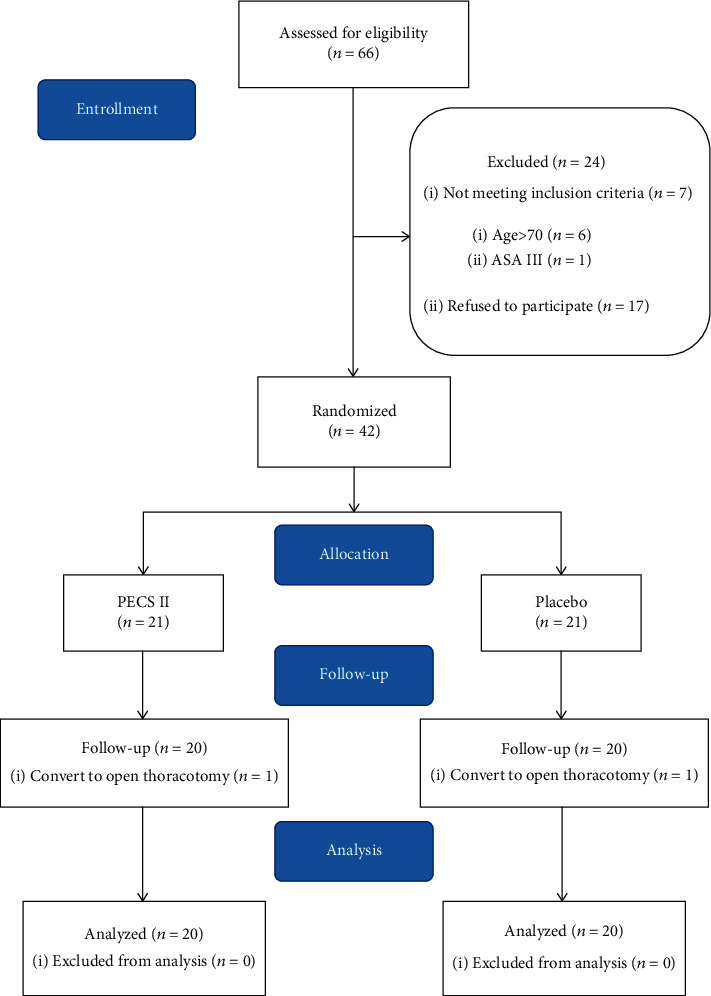
Diagram of this study.

Forty-two subjects were randomly divided into the PECS II group and the placebo group. One subject in each group was eliminated due to the conversion to open surgery during the operation. Finally, 20 subjects in each group were followed up; the results are shown in Figure 1. Besides, the results of the descriptive variables of the two groups were similar (Table 1). Then, there were no significant differences between the PECS II group and the placebo group in operation-related data (Table 2).

Thirty minutes after the block was performed, a pin-prick test was used to analyze the plane of sensory disappearance. Basically, after the completion of the nerve block, the plane of sensory disappearance reached T2 in 18 patients and T6 downward in 12 patients.

The information of perioperative analgesic requirements includes several parts (Table 3). The primary endpoint of this study was the total consumption of fentanyl. Briefly, fentanyl consumption in the PECSII group was significantly lower than that in the placebo group (mean ± SD, 50.25 ± 44.32: 131.50 ± 82.22, *p* < 0.05). The consumption of fentanyl in intravenous analgesia pump within 24 hours was also significantly different between the two groups (mean ± SD, 49.00 ± 44.24: 104.00 ± 72.72, *p* < 0.05).

Intravenous injection of fentanyl was used as a measure of rescue analgesia during the operation and in PACU. Our results showed that the utilization rate of fentanyl during operation in the PECS II group was significantly lower than that in the placebo group (*N* (%), 1 (5) : 12 (60), *p* < 0.05). No patients in the PECS II group required fentanyl for rescue analgesia because their NRS were lower by 4 points in PACU. However, 35% of the patients in the PECS II group received the rescue analgesia in PACU (*p* < 0.05). The analysis of postoperative rescue analgesia rate of patients showed that at 4 hours after operation, the rescue analgesia rate of the placebo group was 75%, while that of the PECS II group was 20%; the difference was statistically significant (*p* < 0.05). At the same time, according to the analysis of postoperative NRS (Table 4), the NRS of the PECS II group was lower than that of the placebo group at 1 h and 4 h after operation, and the difference was statistically significant (*p* < 0.05), but there was no significant difference in NRS between the two groups at 24 hours after operation (*p* > 0.05).

**Figure 2:**
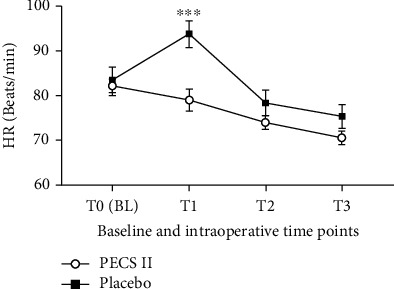
Baseline and intraoperative HR in the PECS II group and the placebo group. Values represent the means and standard deviations. ^∗∗∗^PECS II group vs. placebo group (*p* < 0.0001).

**Figure 3:**
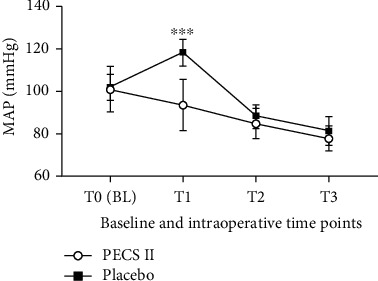
Baseline and intraoperative MAP in the PECS II group and the placebo group. Values represent the means and standard deviations. ^∗∗∗^PECS II group vs. placebo group (*p* < 0.0001).

The results of intraoperative hemodynamic data analysis showed that at T1, the HR and MAP of the PECS II group were significantly lower than those of the placebo group, and the difference was statistically significant (*p* < 0.05), while at T2 and T3, there was no significant difference in HR and MAP between the two groups (*p* > 0.05). Figures 2 and 3 illustrate the above results.

Besides, the results of postoperative adverse reactions showed that the incidence of postoperative nausea and vomiting was 25% in the placebo group and 5% in the PECS II group, and the difference was statistically significant (*p* < 0.05); at the same time, no other adverse reactions were observed.

## 4. Discussion

Compared with the traditional thoracotomy, VATS is associated with less surgical-related trauma, lower local tissue injury, all of which accelerate the recovery of patients. Surprisingly, patients may suffer from severe and persistent pain after VATS [9, 10]. Some studies have suggested that postoperative pain may be associated with tissue injury, implantation of drainage tube, and inflammatory reaction adjacent to chest wall [11, 12]. But no consensus regarding a treatment protocol for acute pain after VATS has been proposed. The most common methods to reduce postoperative pain include epidural analgesia, thoracic paravertebral block (TPVB), and patient-controlled analgesia (PCA) [13]. However, the complications caused by both epidural anesthesia and TPVB are common and serious [14–17].

PECS II is a regional fascial block technique that blocks the thoracic intercostal nerves from T2 to T6, long thoracic and intercostobrachial nerve [8, 18]. Recent studies suggested that PECS II could relieve postoperative pain to a certain extent after mastectomy, reduce the incidence of postoperative chronic pain, and improve the quality of life [19–22]. However, no studies reported on the effect of PECS II after thoracoscopic lobectomy. In the single port thoracoscopic lobectomy, the anterior axillary fourth intercostal space is often selected for resection of the upper lobe of the lung through a 3-5 cm incision or middle and lower lobectomy at the fifth intercostal space [23]. The incision is the primary source of postoperative pain. The early postoperative pain in patients undergoing thoracoscopic lobectomy was mainly concentrated in the incision, while the local chest wall around the incision produced varying degrees of pain. The pain can reach the bottom of the armpit and go down to the next intercostal space of the incision. We speculate that this may be related to the inflammation of the local chest wall [12]. The pain was aggravated after coughing and deep inhalation.

The purpose of this study was to explore the perioperative analgesic effect of PECS II on thoracoscopic lobectomy. The subjects were divided into the placebo group and the PECS II group. The sensory plane and intraoperative hemodynamics after block were observed and recorded, and the postoperative analgesic effect was evaluated at each time point after operation. The main index was the total consumption of fentanyl within 24 hours after operation, and the secondary indexes were NRS, rescue analgesia rate, rescue analgesia time, and so on.

In this study, PECS II block was used as preconditioning before the general anesthesia. Thirty minutes after block, the sensory area in two groups was evaluated by the pin-prick test. A sensory spread to T6 was observed in only 12 patients of the PECS II group. Kulhari et al. [5] analyzed 20 patients who received a PECS II block and found a sensory spread to the T6 segment only in one patient. At the same time, the anesthetic level was terminated at the T5 segment in the rest of them. The authors thought that this might be related to factors such as patients' posture and injection speed. In this experiment, we did not have strict requirements on the injection speed at the experiment design, and we did not achieve the homogenization in terms of local anesthetic injection speed. As for the patients' posture, the anesthesiologist who carried out the nerve block reflected that he did raise the head of the bed slightly at the request of patients. We speculate that the reason why the segment of some patients spread in T6 after the block may be related to the high position of the head.

Besides, we believe that the direction of local anesthetic injection between muscles might be another critical factor affecting the diffusion. Also, our nerve block needle was placed toward the caudal side during the puncture. Hence, the direction of injection and the local anesthetic diffusion were orientated toward the T6 segment, which may be the reason for inconsistent results with that published by Kulhari et al. [5]. Moreover, we noted that there was no high NRS and increased use of opioids because the segment ended at T5.

In our study, only one patient in the PECS II group suffered from nausea and vomiting 6 hours after the operation, and no hematoma was found among the two groups. Ueshima and Otake [24] pointed out that the risk of complications of ultrasound-guided PECS block was low; only eight patients suffered hematoma around the injection area. It is pointed out in the literature that winged scapula syndrome is one of the rare complications of PECS II block [25], but this phenomenon was not found in this study. All these show the safety of PECS II in clinical application.

The NRS and the consumption of fentanyl were significantly higher in the placebo group compared to the PECS II group in 1 hour and 4 hours after the operation (*p* < 0.05). This may be related to factors such as the multiple surgical incisions and placement of a chest tube, which may lead to acute pain. Moreover, we found no difference in T0 between the two groups, but MAP and HR increased significantly in the placebo group at T1. In contrast, MAP and HR in the PECS II group did not increase, which was because the PECS II covered the surgical incision area, making the circulation in the PECS II group more stable. After a significant increase in MAP and HR, the addition of 25 *μ*g fentanyl progressively lowered the blood pressure in 9 patients of the placebo group. By contrast, only one patient in the PECS II group required additional fentanyl during the operation.

In the PECS II group, five patients developed persistent hypotension during the operation, which was resolved after accelerated intravenous fluid infusion, reduced anesthetic infusion, and the administration of vasoactive drugs. Therefore, after preconditioning with PECS II, the consumption of general anesthesia drugs can be largely reduced. In addition, there was no significant difference in MAP and HR between the two groups at T2 and T3. The somatic pain produced at the incision of the chest wall is transmitted by the intercostal nerve, which is then blocked by PECS II. However, the visceral pleura on the surface of the lung is dominated by the autonomic nervous system, which lacks the distribution of sensory nerves and is not sensitive to surgical stimulation, so the stimulation produced during the operation does not seem strong.

In addition, some thought-provoking issues were encountered during this study. The pain that occurred in PACU and 4 hours after the operation was mainly limited to the surgical incision and the surrounding chest wall. Twenty-four hours after the operation, most of the patients felt some pain at the incision. The pain caused by a cough or deep inhalation was often felt not only in the incision itself but also in the medial area of the anterior chest or near the sternum, while some of the patients complained of back pain, which disappeared when the cough stopped. We speculate that this may be related to the position of the drainage tube placed by the surgeon. The implantation of the chest tube can help drainage and even intrathoracic drug delivery. Still, it can also cause complications such as bleeding, pneumothorax, and structural chest injury, injuries to the lung, intercostal artery, diaphragm, and thoracic duct [26, 27]. Malposition of the chest tube may cause Horner's syndrome, arteriovenous fistula formation, and pneumothorax [28]. In this study, all of the patients retained a thoracic drainage tube at the angle from the anterolateral to the medial chest wall. When patients coughed or inhaled deeply, the head end of the drainage tube could stimulate the active parietal pleura. We all know that the intercostal nerve innervates the costal part and neck of the pleural wall layer, the phrenic nerve supplies the diaphragm, and the parietal pleura is the only portion of the pleura that can sense pain. Porcel [29] pointed out that pain has the highest incidence of direct and delayed complications after thoracic drainage tube placement, and the parietal pleura should be anesthetized before insertion of the thoracic drainage tube. Therefore, the pain caused by the stimulation of the drainage tube was more likely to be involved in the formation of pain in the medial region of the anterior chest after the operation.

This study has a few limitations. First, only three time points were used during postoperative follow-up. The reason to choose the 4 h time point was based on our clinical experience. We believe an approach with high frequency was not really practical and challenging to perform, although some previous studies [30, 31] have suggested that this method could be helpful and more accurate. Secondly, not all the operations were performed by the same surgeon, which may produce bias.

## 5. Conclusions

The PECS II block preconditioning can significantly reduce surgical stress, stabilize circulation, prolong the time to first analgesic request, alleviate the pain of surgical incision, and reduce the consumption of opioids without causing severe side effects in patients undergoing thoracoscopic lobectomy.

## Figures and Tables

**Table 1:** The assessment of descriptive variables of two groups.

Characteristics	Groups		*p* value
	PECS II	Placebo	*p*
Age, mean ± SD, y	57.20 ± 8.88	52.75 ± 10.56	>0.05
Sex (*N* (%))			
Male	6 (30)	2 (10)	>0.05
Female	14 (70)	18 (90)	
Height, mean ± SD, cm	160.95 ± 8.33	158.25 ± 6.79	>0.05
Weight, mean ± SD, kg	61.15 ± 8.77	57.93 ± 8.82	>0.05
BMI^∗^, mean ± SD, kg/m^2^	23.77 ± 2.75	23.05 ± 2.07	>0.05
ASA^∗∗^ (*N* (%))			
I	1 (5)	3 (15)	>0.05
II	19 (95)	17 (85)	

All data values are shown as mean ± standard deviations or numbers (percentage). BMI^∗^: body mass index; ASA^∗∗^: American Society of Anesthesiologists.

**Table 2:** Operation-related data of two groups.

Variables	Groups		*p* value
	PECS II	Placebo	*p*
Length of incision^∗^, median (IQR), cm	3.00 (3-4)	3.00 (3-4)	>0.05
Ports (*N* (%))			
Multiple ports	5 (25)	11 (55)	>0.05
Single port	15 (75)	9 (45)	
Procedure (*N* (%))			
Lobotomy	3 (15)	8 (40)	>0.05
Segmentectomy	9 (45)	3 (15)	
Partial resection	8 (40)	9 (45)	
Volume of drainage fluid^∗∗^, mean ± SD, ml	137.50 ± 64.68	119.75 ± 88.62	>0.05
Chest tube insertion, mean ± SD, d	3.35 ± 1.09	3.40 ± 1.64	>0.05
Surgery time, mean ± SD, min	63.25 ± 19.35	78.50 ± 31.00	>0.05
Blood, median (IQR), ml	20.00 (10-30)	30.00 (20-45)	>0.05

Values in this term are presented as mean ± standard deviations, median (interquartile range), or numbers (percentage). Length of incision^∗^: the central operation hole for multiple port VATS in both two groups. Volume of drainage fluid^∗∗^: the fluid in the first 24 h in each group has been collected.

**Table 3:** Perioperative analgesic requirements.

Items	Groups		*p* value
	PECS II	Placebo	*p*
Intraoperative application of fentanyl (*N* (%))			
Yes	1 (5)	12 (60)	<0.05
No	19 (95)	8 (40)	
Application of fentanyl in PACU (*N* (%))			
Yes	0 (0)	7 (35)	>0.05
No	20 (100)	13 (65)	
Time to first analgesia request^∗^ (*N* (%))			
≤4 h	4 (20)	15 (75)	<0.05
>4 h	16 (80)	5 (25)	
Consumption of fentanyl in PCIA, mean ± SD, *μ*g	49.00 ± 44.24	104.00 ± 72.72	<0.05
Total consumption of fentanyl^∗∗^, mean ± SD, *μ*g	50.25 ± 44.32	131.50 ± 82.22	<0.05

All data values are shown as mean (standard deviations) or numbers (percentage). Time to first analgesia request^∗^: the first time that patients received the treatment of fentanyl after operation. Total consumption of fentanyl^∗∗^: included the dose of fentanyl used for rescue analgesia intraoperatively and within the PACU and the dose of fentanyl consumed in the intravenous analgesia pump within 24 hours.

**Table 4:** Assessment of numerical rating scale (NRS).

Time points	Groups		*p* value
	PECS II	Placebo	*p*
1 (PACU)^∗^, mean ± SD, h			
Cough^∗∗^	2.80 ± 1.01	4.60 ± 1.05	<0.05
Rest	1.65 ± 0.75	3.20 ± 0.70	<0.05
4, mean ± SD, h			
Cough	3.15 ± 0.75	4.50 ± 1.00	<0.05
Rest	2.10 ± 0.72	3.10 ± 0.85	<0.05
24, mean ± SD, h			
Cough	2.55 ± 0.83	3.00 ± 0.86	>0.05
Rest	1.55 ± 0.83	1.75 ± 0.85	>0.05

All the data values are presented as mean (standard deviations). 1 h (PACU)^∗^: evaluation of NRS at PACU is regarded as the time point of 1 h after operation. Cough^∗∗^: dynamic NRS at 1, 4, and 24 h after operation are induced by coughing.

## Data Availability

It can be provided by the author via email.

## Supplementary Materials

Supplementary materials Patients' satisfaction was evaluated with a score of 0-10, with 0 as the most dissatisfied and 10 as the most satisfied. We have added this part in the attachment provided.
