# Supplementary material for: Pretreatment with Pectoral Nerve Block II Is Effective for Reducing Pain in Patients Undergoing Thoracoscopic Lobectomy: A Randomized, Double-Blind, Placebo-Controlled Trial
Source: Biomed Res Int. 2021 Apr 16;2021:6693221. doi: 10.1155/2021/6693221 (PMC8068546; doi:10.1155/2021/6693221)
Supplement: Supplementary materials — Patients' satisfaction was evaluated with a score of 0-10, with 0 as the most dissatisfied and 10 as the most satisfied. We have added this part in the attachment provided. [file 6693221.f1.docx]

Supplementary material

| *Incidence of postoperative nausea and vomiting and patient satisfaction* | | | |
| --- | --- | --- | --- |
| variables | Groups | | P-value |
|  | PECSII | Placebo | P |
| Nausea and vomiting [N (%)] | 1 (5) | 5 (25) | <0.05 |
| *Satisfaction, mean ± SD | 7.65±2.03 | 3.04±1.77 | <0.05 |

*Satisfaction: Patients' satisfaction was evaluated with a score of 0-10, with 0 as the most dissatisfied and 10 as the most satisfied.
